# Dextran Sodium Sulfate (DSS) Induces Colitis in Mice by Forming Nano-Lipocomplexes with Medium-Chain-Length Fatty Acids in the Colon

**DOI:** 10.1371/journal.pone.0032084

**Published:** 2012-03-09

**Authors:** Hamed Laroui, Sarah A. Ingersoll, Hong Chun Liu, Mark T. Baker, Saravanan Ayyadurai, Moiz A. Charania, Famina Laroui, Yutao Yan, Shanthi V. Sitaraman, Didier Merlin

**Affiliations:** 1 Department of Biology, Center Diagnostics and Therapeutics, Georgia State University, Atlanta, Georgia, United States of America; 2 Veterans Affairs Medical Center, Decatur, Georgia, United States of America; 3 Department of Medicine, Division of Digestive Diseases, Emory University, Atlanta, Georgia, United States of America; 4 Department of Gastroenterology, Zhongshan Hospital, Fudan University, Shanghai, China; Ulm University, Germany

## Abstract

Inflammatory bowel diseases (IBDs), primarily ulcerative colitis and Crohn's disease, are inflammatory disorders caused by multiple factors. Research on IBD has often used the dextran sodium sulfate (DSS)-induced colitis mouse model. DSS induces *in vivo* but not *in vitro* intestinal inflammation. In addition, no DSS-associated molecule (free glucose, sodium sulfate solution, free dextran) induces *in vitro* or *in vivo* intestinal inflammation. We find that DSS but not dextran associated molecules established linkages with medium-chain-length fatty acids (MCFAs), such as dodecanoate, that are present in the colonic lumen. DSS complexed to MCFAs forms nanometer-sized vesicles ∼200 nm in diameter that can fuse with colonocyte membranes. The arrival of nanometer-sized DSS/MCFA vesicles in the cytoplasm may activate intestinal inflammatory signaling pathways. We also show that the inflammatory activity of DSS is mediated by the dextran moieties. The deleterious effect of DSS is localized principally in the distal colon, therefore it will be important to chemically modify DSS to develop materials beneficial to the colon without affecting colon-targeting specificity.

## Introduction

Inflammatory bowel diseases (IBDs), principally ulcerative colitis (UC) and Crohn's disease (CD), are inflammatory disorders of the gastrointestinal tract caused by multiple genetic and environmental factors [1], [2], [3]. Various models of experimental IBD have been developed to investigate pathogenesis and to improve treatment options such as gene knockout (KO) models:interleukin (IL)-2/IL-2 receptor-alpha [4], IL-10 [5], T cell receptor [6], Tumor necrosis factor (TNF)-3′ untranslated region (UTR) [7] or transgenic models: IL-17 [8], HLA B27 [9]. Most commonly, experimental colitis is induced by the heparin-like polysaccharide DSS; this model is simple and affords a high degree of uniformity and reproducibility of most lesions in the distal colon [10], [11]. By first interfering with intestinal barrier function, and next stimulating local inflammation, DSS is often used to induce a form of mouse colitis that mimics the clinical and histological features of IBDs that have characteristics of UC [12], [13], [14]. The typical features of colitis appear on day 3 and are maximally expressed by day 7 [15]. Notably, expression of pro-inflammatory cytokines and chemokines (IL-1, IL-6, KC, TNF-α, and Interferon-γ) are upregulated [15], [16] whereas synthesis of anti-inflammatory cytokines, such as IL-10, is downregulated [16], [17], [18], [19]. Other parameters such as decreases in body weight and colon length, elevation in myeloperoxidase level (suggestive of neutrophil infiltration into the epithelium), and higher histological and endoscopic scores characterize murine colitis [20], [21], [22]. DSS induces colitis, but the mechanism of action remains unknown. Miyazawa et al. [23] showed that DSS caused disruption of biological mechanisms (such as inhibitory effects on reverse transcriptase activities that affect major cellular functions), competing with poly(U) to this end [23]. Previously, it was shown that dextran sulfate inhibited ribonuclease action [24], [25]. Other natural and synthetic polyanionic polymers play important roles in establishing the association of mRNA with ribosomes and can disturb mRNA translation [23]. But the mechanism of how DSS penetrates the cell is unknown as it could be through passive or active uptake by the cell via a specific receptor, or DSS could penetrate the cell after complexation with another molecular form (such as polycationic forms). Other studies have shown that DSS induced significant macrophage infiltration into the epithelium of the colon [26]. Our study attempted to correlate this last hypothesis by increasing medium-chain-length fatty acids (MCFAs) in the mouse colon. MCFAs are increased by a high fat diet. The role of MCFAs in inflammation is not clear as the size of the carbon chain is intermediate between small and long chains. The sodium salts of several MCFAs, particularly capric (C10) and lauric (C12) acids, have been shown to increase rectal drug absorption, presumably by causing alterations in intestinal tight junctions (TJ) barrier function. C10 has also been shown to lead to profound alterations in the barrier function of the TJ and has been investigated as an agent to enhance viral-mediated a gene transfer [27]. These agents are attractive as potential therapies to enhance absorption of gene transfer vectors due to the rapid onset of action (within minutes) and their relatively rapid recovery (within hours) after treatment [28], [29], [30], [31] [27].

Although MCFAs have been widely investigated as agents to increase the delivery of therapeutic agents, relatively little is known regarding their primary mechanism of action. The ability of intercellular TJs to function as a barrier to the diffusion of macromolecules is dynamically regulated by numerous intracellular signals and the permeability properties of the TJ vary in response to changes in physiological state. Therefore, agents that regulate the TJ likely do so by direct or indirect actions on intracellular signals and/or the protein components of the TJ.

It has been extensively shown that short chain fatty acids, like butyrate, attenuate inflammation in DSS-induced colitis [32], [33], [34], [35]. Conversely, long chain fatty acids, like palmitic acid (C16) or palmitic acid (monosaturated or polysaturated forms) have been shown to promote inflammation [26]. Kim et al. have shown that corn oil, mainly constituted with the long chain fatty acid cited above, have a significant effect on the aggravation of inflammation related colorectal cancer [26].

In the present study we investigated the mechanism of DSS action in the distal colon using *in vitro* and *in vivo* approaches.

## Materials and Methods

### Animal studies

All studies were performed in accordance with the Institutional Animal Care and Use Committee at Emory University (Atlanta, GA). All procedures were approved and are registered in the protocol IACUC ID: 156–2008, approval date 7/8/2010 to 7/8/2011. Strains, ages, and the number of animalsfollow the established protocol.

Female C57BL/6 mice (aged 8 weeks, weight 18–22 g, Jackson Laboratories, Bar Harbor, ME) used for this study were group-housed under controlled temperature (25°C) and photoperiod (12∶12-hour light–dark cycle) conditions, and given unrestricted access to standard diet and tap water (or specified drinking solution). Mice were allowed to acclimate to these conditions for at least 7 days before inclusion in experiments.

High fat diet (60% kCal of fat, 20% kCal of protein, and 20% kCal of carbohydrate) and control diet (10% kCal of fat, 20% kCal of protein, and 70% kCal of carbohydrate) were purchased from Research Diets (New Brunswick, NJ). Supplementation of fat is provided by soybean oil (10%) and lard (90%).

### Histology

Distal colonic sections were fixed in 10% formalin and embedded in paraffin. 5-µm sections were stained with H&E. Images were acquired using a Zeiss Axioskop 2 plus microscope (Carl Zeiss MicroImaging) equipped with an AxioCam MRc5 CCD camera (Carl Zeiss).

### Histological score assessment of colitis

H&E-stained colonic sections were coded for blind microscopic assessment of inflammation (i.e., DSS-induced colitis). Histological scoring was based on 3 parameters. Severity of inflammation was scored as follows: 0, rare inflammatory cells in the lamina propria; 1, increased numbers of granulocytes in the lamina propria; 2, confluence of inflammatory cells extending into the submucosa; 3, transmural extension of the inflammatory infiltrate. Crypt damage was scored as follows: 0, intact crypts; 1, loss of the basal one-third; 2, loss of the basal two-thirds; 3, entire crypt loss; 4, change of epithelial surface with erosion; 5, confluent erosion. Ulceration was scored as follows: 0, absence of ulcer; 1, 1 or 2 foci of ulcerations; 2, 3 or 4 foci of ulcerations; 3, confluent or extensive ulceration. Values were added to give a maximal histological score of 11.

### Endoscopic assessment of colitis

Direct visualization of DSS-induced colonic mucosal damage in vivo was performed using the Coloview (Karl Storz Veterinary Endoscopy, Tuttlingen, Germany). Mice were supplied with food and water until the endoscopy was performed. Mice were anesthetized with 1.5 to 2% isoflurane and 3 cm of the colon proximal to the anus was visualized after inflation of the colon with air. The endoscopic damage score was determined using a previously described scoring method with one modification: assessment of colon translucency (0–3 points), presence of fibrin attached to the bowel wall (0–3 points), granular aspect of the mucosa (0–3 points), morphology of the vascular pattern (0–3 points), and stool characteristic (normal to diarrhea; 0–3 points) [21]. Since this scoring method did not include assessment for the presence of blood in the lumen, we added this parameter (0 points: no blood; 1 point: slight bleeding; 2 points: frank bleeding) to generate a range in total score from 0–17 points.

### Pro-inflammatory chemokine and cytokine analysis

Total RNA was extracted using TRIzol reagent (Invitrogen, Carlsbad, CA) and reverse-transcribed using the cDNA Synthesis kit (Fermentas, Glen Burnie, MD). RT-PCR was performed using the GeneJET Fast PCR kit (Fermentas, Glen Burnie, MD). Real-time RT-PCR was performed using an iCycler (Bio-Rad, Hercules, CA). Briefly, cDNA was amplified by 40 cycles of 95°C for 15 s and 60°C for 1 min, using the iQ SYBR Green Supermix system (Biorad, Hercules, CA) and the following specific primers:

KC sense 5′-TTGTGCGAAAAGAAGTGCAG-3′, KC antisense 5′-TACAAACACAGCCTCCCACA-3′,

IL-1β sense 5′-TCGCTCAGGGTCACAAGAAA-3′, IL-1β antisense 5′-CATCAGAGGCAAGGAGGAAAA C-3′;

TNF-α sense 5′-AGGCTGCCCCGACTACGT-3′, TNF-α antisense 5′-GACTTTCTCCTGGTATGAGATAGCAAA-3′;

IL-6 sense 5′-ACAAGTCGGAGGCTTAATTACACAT-3′, IL-6 antisense 5′-TTGCCATTGCACAACTCTTTT C-3′;

IFN-γ sense 5′-CAGCAACAGCAAGGCGAAA-3′, IFN-γ antisense 5′-CTGGACCTGTGGGTTGTTGAC-3′;

IL-10 sense 5′-ACCTGGTAGAAGTGATGCCCCAGGCA-3′ and 5′-CTATGCAGTTGATGAAGATGTCAAA-3′

18S sense 5′-CCCCTCGATGACTTTAGCTGAGTGT-3′, 18S antisense 5′-CGCCGGTCC AAGAATTTCACCTCT-3′;

mouse 36B4 sense 5′-TCCAGGCTTTGGGCATCA-3′, mouse 36B4 antisense 5′-CTTTATCAGCTGCACATCACTCAGA-3′.

18S and 36B4 were used as housekeeping genes. Fold-induction was calculated using the Ct method, ΔΔCT = (*C*t_Target_-*C*t_housekeeping_)_infected_ − (*C*t_Target_-*C*t_housekeeping_)_uninfected_, and the final data were derived from 2^−ΔΔCT^.

### Myeloperoxidase (MPO) assay

Colonic tissue samples were homogenized in ice-cold potassium phosphate buffer (50 mM K2HPO4 and 50 mM KH2PO4, pH 6.0) containing 0.5% hexadecyltrimethylammonium bromide (Sigma). The homogenates were then sonicated, freeze-thawed three times, and centrifuged at 17,500 rcf for 15 min. Supernatants (20 µl) or MPO standard were added to 1 mg/mL *o*-dianisidine hydrochloride (Sigma) and 0.0005% H_2_O_2_, and the change in absorbance at 450 nm was measured. One unit of MPO activity was defined as the amount that degraded 1 µmol peroxidase per minute. The results were expressed as relative MPO activity compared to water-treated mice (normalized to 1).

### DSS-induced colitis

Colitis was induced by 3% (w/v) dextran sodium sulfate (DSS; molecular weight 42 kDa; ICN Biochemicals, Aurora, OH) added to the drinking water. Colonic inflammation was assessed 7 to 8 days after DSS treatment. Twelve mice were included in each group. To evaluate the effects of each drinking solution on induced colitis, the mice were given a bottle of each solution with equivalent number of moles of the studied molecules: DSS 30 g/L, dextran 14.9 g/L, glucose 17.33 g/L, and sulfate 25.99 g/L. The concentrations were obtained by calculating the concentration of dextran sodium sulfate: 6.98×10^−4^ mol.L^−1^. The DSS supplier (MP Biomedicals) verified that the sulfate (SO3H) substitution rate was 1.9 groups per glycosyl residue of DSS. We approximated 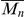 = 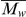 = 43000 g/mol and calculated the degree of polymerization (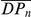) of DSS:


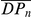
 = 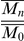



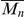
 = numbered molecular mass of DSS


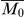
 = Molecular mass of a single glycosyl residue grafted with 1.9 sulfate group


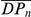
 = 43000/310.6 = 138

In one polymer chain of DSS, there are 138 glycosyl residues. Equivalent concentrations for DSS associated molecules have to be calculated according to (DSS = 138 residues = 30 g/L; dextran = 138 residues = 14.9 g/L; sodium sulfate = 138*1.9 = 262.2 residues = 25.99 g/L; glucose = 138 residues = 17.33 g/L).

### Osmolarity of mice feces

Feces was collected and dried in an incubator at 37°C (n = 6 per drinking solution treatment). 50 mg of feces was dissolved in 1 mL of NaCl (0.15 M). Osmolarity of water treated mice was set to 1 arbitrary unit (AU) as negative control. All osmolarities were then measured and calculated as relative osmolarity (Osmolarity of sample feces/Osmolarity of water-treated mice feces). Osmolarity measurement was performed by an osmometer (Osmette III, Precision Systems, Natick, MA).

### Epithelium resistance measurement by Electric Cell-substrate Impedance Sensing (ECIS)

For ECIS, cell-attachment assays were performed using ECIS technology (Applied BioPhysics, Troy, NY) [36]. The ECIS model 1600R (Applied BioPhysics) was used for these experiments. The measurement system consists of an 8-well culture dish (ECIS 8W1E plate), the surface of which is seeded with cell cultures. Each well contains a small, active electrode (area = 5.10^−4^ cm^2^) and a large counter electrode (area = 0.15 cm^2^) on the bottom of each well. A lock-in amplifier, with an internal oscillator, relays a signal to switch between the different wells, and a personal computer controls the measurement and stores the data. Attachment and spreading of cells on the electrode surface change the impedance in such a way that morphological information about the attached cells can be inferred. Caco2-BBE cells (1 million cells per mL) were seeded in ECIS 8W1E plates in DMEM (Invitrogen) supplemented with 10% (v/v) heat-inactivated fetal bovine serum (Invitrogen) and 1.5 µg/mL plasmocin (Invitrogen). Once cells reached confluence, the different solutions were added at a concentration given in the results section. Controls in DMEM medium cell cultures were used for each experiment. Basal resistance measurements were performed using the ideal frequency for Caco2-BBE cells, 500 Hz, and a voltage of 1 V.

### Dextran-loaded nanoparticles synthesis

Poly (D,L-lactide) (PLA) was purchased from Sigma-Aldrich (St Louis, MO) (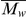 of 75–120 kg/mol). The nanoparticles were produced by the double emulsion/solvent evaporation procedure previously described [37] for the elaboration of polyvinylic alcohol (PVA)-covered PLA nanoparticles. This method involves use of an amphiphilic molecule as the surfactant of the secondary emulsion; however, in the present study, PVA was used, which was purchased from Aldrich (Milwaukee, WI) (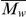 = 13,000–23,000 g/mol, hydroxylated at 87–89%). Typically, a primary water in oil emulsion (w/o) was prepared by mixing an organic phase (4 mL dichloromethane) containing PLA (25 g/L) with an internal aqueous phase (400 µL). The emulsion is stabilized by the bovine serum albumin (BSA; 50 g/L) of the internal aqueous phase. The internal phase also contains, or does not contain, dextran (2.5 g/L, 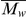 = 40 kDa). The mixture was stirred for 2 min with a Vortex mixer (Maxi Mix II, Thermolyne, USA), then sonicated (2 min, power 6, 50% active cycle, in an ice bath) using a Sonifier 450 (Branson, USA). This primary emulsion was poured into a second aqueous phase (8 mL) (external aqueous phase) containing an amphiphilic molecule (PVA 20 g/L). The water in oil in water emulsion (w/o/w) was then obtained by sonication (same conditions as those used for the primary emulsion). This double emulsion was then transferred to an aqueous 10^−2^ M NaNO_3_ dispersing phase (40 mL) and stirred for 5 min. The organic solvent was evaporated under stirring and ambient air conditions and the collected solid nanospheres were resuspended in water then centrifuged again to remove the excess PVA. This purification procedure was repeated twice. The final suspension was then freeze-dried.

### Particle size measurement

Diameters (nm) of NPs were measured by light scattering using 90 Plus/BI-MAS (Multi angle particle sizing, Brookhaven Instruments Corporation, Holtsville, NY, USA). The average and standard deviations of the diameters (nm) were calculated using 3 runs. Each run is an average of 10 measurements.

### The effect of encapsulated dextran-loaded NPs on in vivo inflammation

To deliver the dextran-loaded NPs to C57BL/6 mice colonic lumen, we encapsulated them into a biomaterial comprised of alginate and chitosan at a ratio of 7/3 (wt/wt). We and others have previously shown that the biomaterial collapses in intestinal solution at pH 5 or 6, which is the colonic pH under inflamed and non-inflamed states [38], [39], [40]. C57BL/6 mice (8 per group) were gavaged daily for 4 days with encapsulated dextran-loaded or empty NPs as a negative control.

### Cell Culture

Caco2-BBE cells were cultured to confluency in 75-cm^2^ flasks at 37°C in a humidified atmosphere containing 5% CO_2_. The culture medium used was DMEM/Ham's F-12 medium (Invitrogen, USA) supplemented with L-glutamine (2 mM), penicillin (100 units/mL), streptomycin (100 µg/mL), and heat-inactivated fetal calf serum (10%) (Invitrogen, USA).

### Statistical analysis

Data are presented as average values and standard deviations from experiments performed in triplicate (n = 3), except *in vivo* experiments (n = 8). ANOVA tests were performed to obtain statistical comparisons between samples.

## Results

### Characterization of DSS-induced colitis in mice

**Figure 1:**
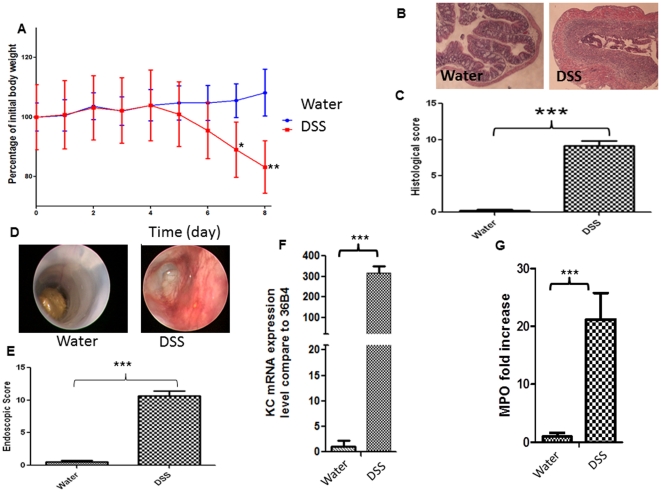
DSS induces colitis. (A) Mouse body weight changes during DSS treatment compared to that of control animals (drinking water). C57BL/6 mice were exposed to 3% (w/v) DSS in the drinking water for the indicated numbers of days. Body weight changes are shown as means±SEMs. * P<0.05, ** P<0.01. (B) Hematoxylin-stained colonic sections of mice treated with DSS; the mice were sacrificed on day 8. (C) Associated histological scores. *** P<0.001. (D) Macroscopic inflammation was assessed using a mouse colonoscope. Photographs were obtained on the day of sacrifice and (E) an endoscopic score was calculated. *** P<0.001. (F) KC mRNA expression was measured in test and control animals. *** P<0.001. (G) Determination of MPO enzymatic activity as an index of neutrophil infiltration into injured tissue. Results are expressed as MPO –fold increases compared to those of control mice and represent means±SEMs of three independent determinations. *** P<0.01.

The DSS-induced colitis murine model is commonly used to address the pathogenesis of IBD [15], [20], [41] and to test the efficacy of therapies such as nanotechnology-based drug release systems [39], [40], [42]. C57BL/6 mice were given 3% (w/v) DSS in drinking water for 8 days. Significant weight loss was evident in the DSS-treated group compared to the negative control (Figure 1A). About 20% of initial weight was lost after 8 days of DSS treatment (Figure 1A). During this treatment, inflammation was enhanced, as shown by rises in the extent of diarrhea and rectal bleeding. Colonic inflammation was also examined by histology (Figure 1B); hematoxylin-stained day 8 colon sections of DSS-treated mice showed severe lesions throughout the mucosa, alteration of epithelial structure, high-level neutrophil and lymphocyte infiltration into the mucosal and submucosal areas, and loss of crypts (Figure 1B). All alterations observed were combined to obtain histological scores (Figure 1C). The score calculated after DSS treatment was significantly higher than that of control mice; 9.2±1.5 versus 0.2±0.4, respectively. Histological observations were confirmed by macroscopic inflammation assessment using a mouse colonoscope. Photographs were obtained on the day of sacrifice (thus after 8 days of DSS treatment; Figure 1D); the colon was inflamed. Colon photographs after DSS treatment revealed bloody stools, diarrhea, ulcers, and mucosal inflammation; control animals had healthy mucosa. Figure 1E shows the endoscopic scores obtained upon analysis of the photographs; DSS-treated mice scored significantly higher than did control animals (10.6±1.3 versus 0.7±0.8). These macroscopic observations were confirmed by estimation of biological parameters relevant to inflammation. We measured expression of KC, a chemokine. DSS significantly increases KC production [43], [44], [45]. Expression of mRNA encoding KC was measured in DSS-treated and control mice. The extent of KC mRNA expression was 300-fold higher in DSS-treated animals compared to control animals (Figure 1F). MPO enzymatic activity (an index of neutrophil infiltration into the colonic mucosa) increased 20-fold in DSS-treated mice compared to controls (Figure 1G). The mouse DSS-induced colitis model can thus be used to address the pathogenesis of IBD.

### Specificity of DSS for induction of colitis

**Figure 2:**
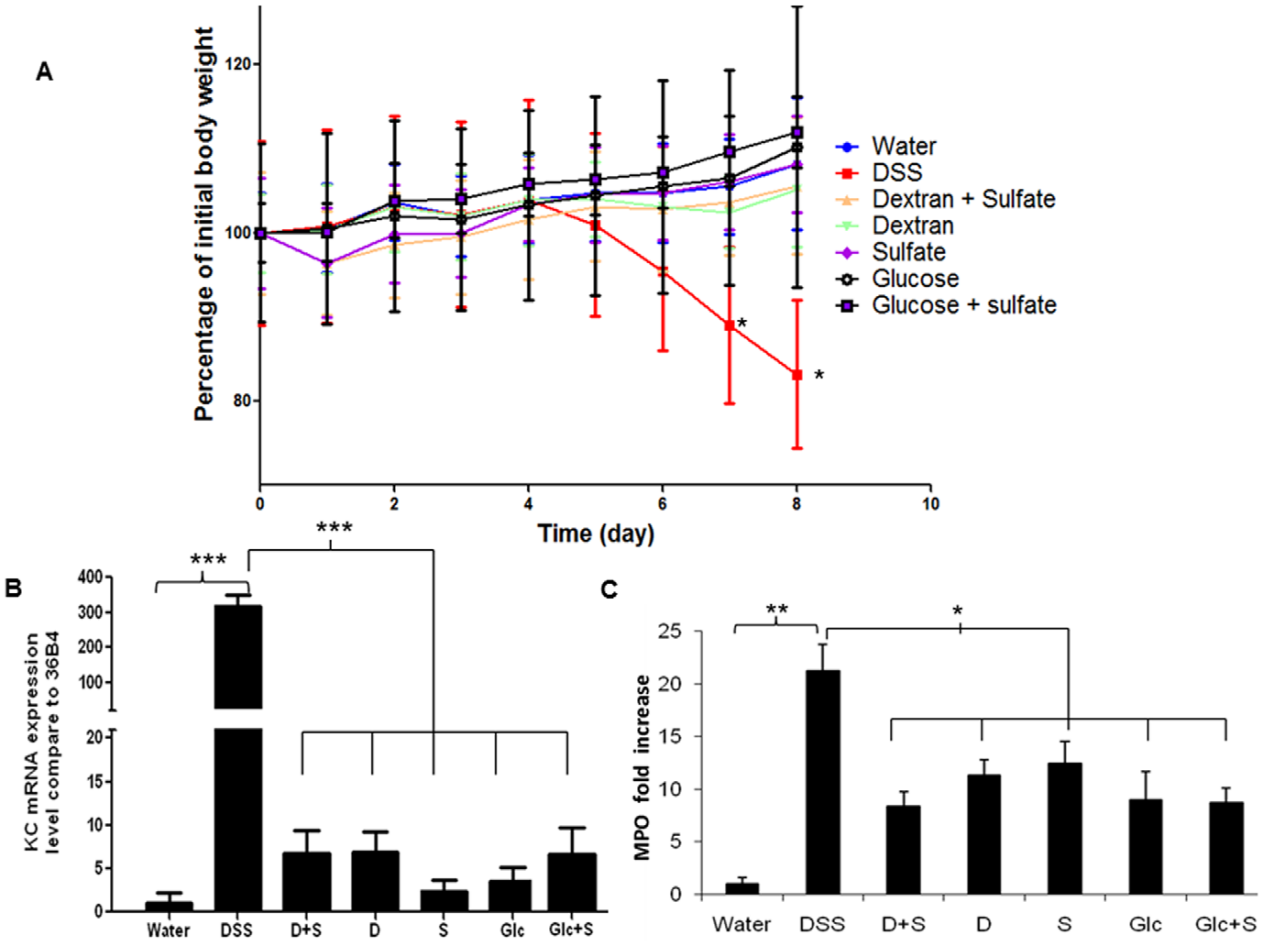
DSS but not DSS-associated molecules induce colitis. (A) Mouse body weight changes during consumption of water, DSS (3% w/v), dextran (1.49% w/v) in sulfate solution (2.6% w/v), dextran (1.49% w/v), sulfate solution (2.6% w/v), glucose (1.73% w/v), and glucose (1.73% w/v) in sulfate solution (2.6% w/v). C57BL/6 mice were given the various solutions as drinking water for the indicated numbers of days. Body weight changes are depicted as means±SEMs for each group. * P<0.05. (B) DSS significantly increased production of chemokine KC mRNA compared to the rises seen when DSS-associated molecules were given. KC mRNA expression was measured via qRT-PCR of extracts of colonic cells. *** P<0.001. (C) Determination of MPO enzymatic activity as an index of neutrophil infiltration into injured tissue. (D) Macroscopic observations of colons of mice treated with DSS-associated molecules [DSS = dextran sodium sulfate, D+S = dextran (1.49% w/v) in sulfate solution (2.6% w/v), D = dextran (1.49% w/v), S = sulfate solution (2.6% w/v), and Glc+S = glucose (1.73% w/v) in sulfate solution (2.6% w/v)] were made using a mouse colonoscope. Photographs were obtained from all treatment groups on the day of sacrifice. * P<0.05, ** P<0.01.

**Figure 3:**
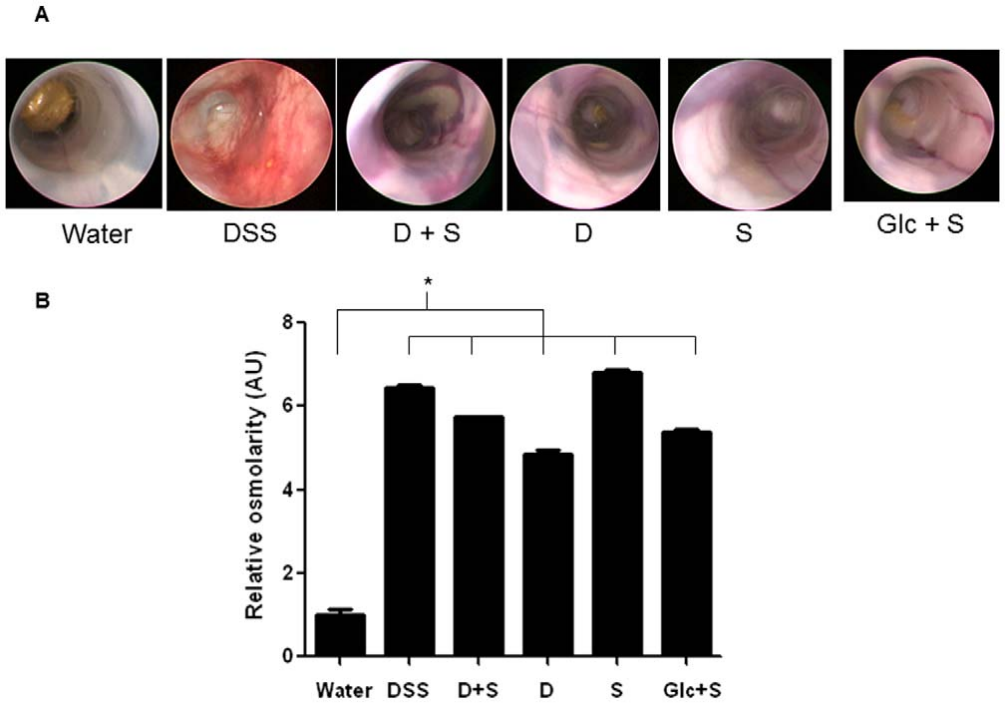
DSS and DSS-associated molecules affect luminal intestinal osmolarity. (A) Macroscopic observations of the effects of DSS-associated molecules [DSS = dextran sodium sulfate, D+S = dextran (1.49% w/v) in sulfate solution (2.6% w/v), D = dextran (1.49% w/v), S = sulfate solution (2.6% w/v), and Glc+S = glucose (1.73% w/v) in sulfate solution (2.6% w/v)] on colonic epithelium were assessed using a mouse colonoscope. Photographs were obtained from all treatment groups on the day of sacrifice. (B) Fecal relative osmolarity values (means±SEM, ANOVA statistical test, * P<0.05).

We explored how DSS, a negatively charged polymer of glucose with engrafted sulfate groups, induced colitis in mice. As DSS is composed of successive glucose units substituted with sulfur groups (−SO_3_H), we decided to test each DSS component separately. Thus, we explored the effects of dextran dissolved in water, dextran dissolved in a sulfate salt solution, sulfate salt dissolved in water, glucose dissolved in water, and glucose dissolved in a sulfate salt solution. To be consistent, the ratios of glucose, dextran, or sulfate molecules were identical to those in DSS. All concentrations were based on a 3% (w/v) DSS solution (mol. wt. 42 kDa; 17% by weight of engrafted sulfate groups). As shown in Figure 2A, DSS given in drinking water for 8 days increased colonic inflammation, as measured by body weight loss, however there was no observable inflammation in the other treated groups. Also, we did not observe diarrhea or rectal bleeding in mice treated with DSS-associated molecules (data not shown). DSS induction of KC-encoding mRNA was 300-fold higher than the control value whereas the maximum increase afforded by ingestion of DSS-associated molecules did not exceed 7-fold (Figure 2B). Determination of MPO enzymatic activity as an index of neutrophil infiltration into intestinal mucosa revealed a 20-fold increase in MPO activity in DSS-treated mice compared to controls (Figure 2C). A 10-fold increase in MPO level after treatment with DSS-associated molecules was observed compared to the control value (Figure 2C). Macroscopic features of inflammation were assessed using a mouse colonoscope. In contrast to DSS, DSS-associated molecules did not induce the common characteristics of an inflamed colon (Figure 3A). However, whereas intestinal mucosa treated with DSS-associated molecules was intact, hemorrhagic vessels were observed in the submucosal layer after ingestion of all DSS-associated molecules (Figure 3A). We hypothesize that a local increase in osmolarity may affect the integrity of blood vessels (Figure 3A). To verify this hypothesis, we measured the osmolarity of feces. We found that mice treated with DSS and DSS-associated molecules had higher fecal osmolarity than did control animals (Figure 3B). This is consistent with the observed vessel degradation and submucosal bleeding in such animals, and the subsequent increase in neutrophil infiltration into the mucosa, as shown by MPO measurement (Figure 2C). Together, the data show that DSS is specific in terms of colitis induction in mice. No DSS-associated molecule tested induced colitis after 1 week of treatment. However, we suggest that DSS-associated molecules can cause development of hemorrhagic vessels mainly because of the change of colonic osmolarity in these groups.

### DSS and DSS-associated molecules do not affect the electrical resistance of Caco2-BBE cell monolayers

**Figure 4:**
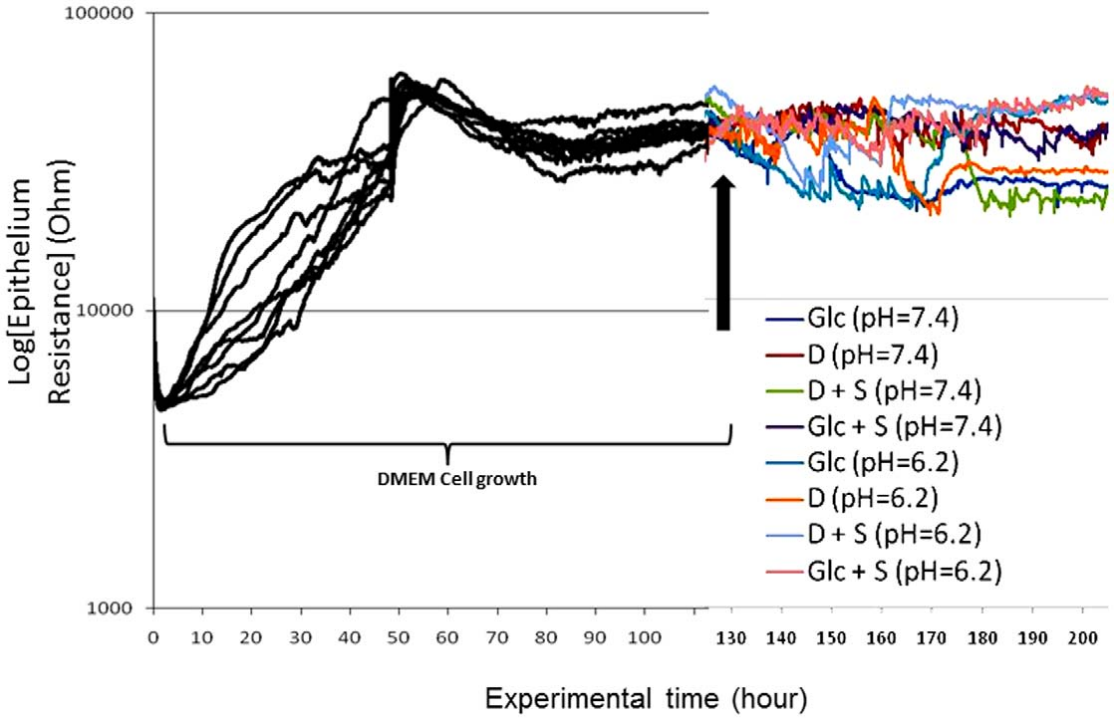
DSS-associated molecules do not affect the resistance of Caco2-BBE monolayers. Confluent cultures of Caco2-BBE cells were obtained after 48 h (dark lines) and epithelial resistance was assessed by conduct of continuous resistance measurements at pH 7.4 (cellular pH) and pH 6.2 (colonic pH). Resistance (Ohm) of Caco2-BBE cells was measured at Ω = 500 Hz and V = 1 V by electrical impedance sensing method (ECIS) on ECIS 8W1E electrodes. After confluence (colored lines), DMEM-based solutions containing glucose 17.33 g/L (Glc), dextran 14.9 g/L (D), dextran 14.9 g/L and sodium sulfate 25.99 g/L (D+S) and glucose 17.33 g/L and sulfate 25.99 g/L (Glc+S) were added at both pH. Data represent means of n = 3/condition.

As shown in Figure 4, using ECIS, we obtained confluent Caco2-BBE monolayer cells as assessed by attainment of a plateau with a high resistance value (R = 36,000 Ω; dark lines of Figure 4).

**Figure 5:**
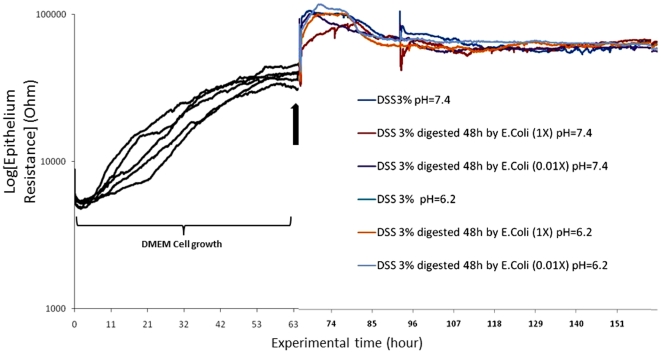
DSS and DSS sub-products obtained when DSS is digested by *E. coli* do not affect the resistance of Caco2-BBE monolayers. Confluent cultures of Caco2-BBE cells were obtained after 48 h (dark lines) and epithelial resistance was assessed by conduct of continuous resistance measurements at pH 7.4 (cellular pH) and pH 6.2 (colonic pH). Resistance (Ohm) of Caco2-BBE cells was measured at Ω = 500 Hz and V = 1 V by electrical impedance sensing method (ECIS) on ECIS 8W1E electrodes. After confluence (colored lines), DMEM-based solutions containing potential sub-products of the digestion of DSS (3%) by Escherichia coli at high concentration (4.10^11^ CFU/mL, 1×) or diluted (4.10^9^ CFU/mL, 0.01×) were added at both pH. Data represent means of n = 3/condition.

After adding the DMEM-based solution containing glucose 17.33 g/L (Glc), dextran 14.9 g/L (D), dextran 14.9 g/L and sodium sulfate 25.99 g/L (D+S) and glucose 17.33 g/L and sulfate 25.99 g/L (Glc+S) to the confluent Caco2-BBE monolayer of cells, the resistance plateau values (R = 36 kΩ) did not decrease (colored lines of Figure 4). Interestingly, no DSS-associated molecules affected monolayer resistance when the extracellular medium was at pH 7.4 or pH 6.2 (Figure 4). This shows that the *in vivo* ability of DSS, compared to DSS-associated molecules, to induce colitis, is lost *in vitro*. This suggests that some permissive *in vivo* “factor” must be present to allow DSS to induce colitis. The lack of a DSS effect on resistance *in vitro* suggests that DSS does not interact with an intestinal epithelial receptor (Figure 5).

### DSS sub-products produced by Escherichia coli action do not affect Caco2-BBE cell monolayer resistance

When DSS is given to mice, DSS may interact with colonic bacteria. We considered that DSS might be chemically modified by such bacteria, producing a sub-product affecting intestinal barrier function. To test this hypothesis, we supplemented bacterial medium with 3% (w/v) DSS and grew *Escherichia coli* in the medium for 48 h at 37°C at a high concentration (saturated growth medium, 4.10^11^ CFU/mL, 1×) and at diluted concentration (4.10^9^ CFU/mL, 0.01×). The culture supernatant was collected and the pH adjusted to pH 7.4 or pH 6.2.

As shown in Figure 5, using ECIS, we obtained confluent Caco2-BBE monolayer cells as assessed by attainment of a plateau with a high resistance value (R = 42,000 Ω; dark lines of Figure 5).

Next, supernatant samples were added to confluent Caco2-BBE monolayers and online measurement of epithelial resistance was performed using ECIS (colored lines of Figure 5). As shown in Figure 5, supernatant at pH 7.4 or 6.2, containing DSS and presumably DSS sub-products, did not affect Caco2-BBE monolayer resistance as the value of the resistance did not change. This showed that bacteria are not the permissive “factor” mediating the *in vivo* specificity of DSS in induction of colitis.

### A high-fat diet increases the efficacy by which DSS induces colitis in mice

**Figure 6:**
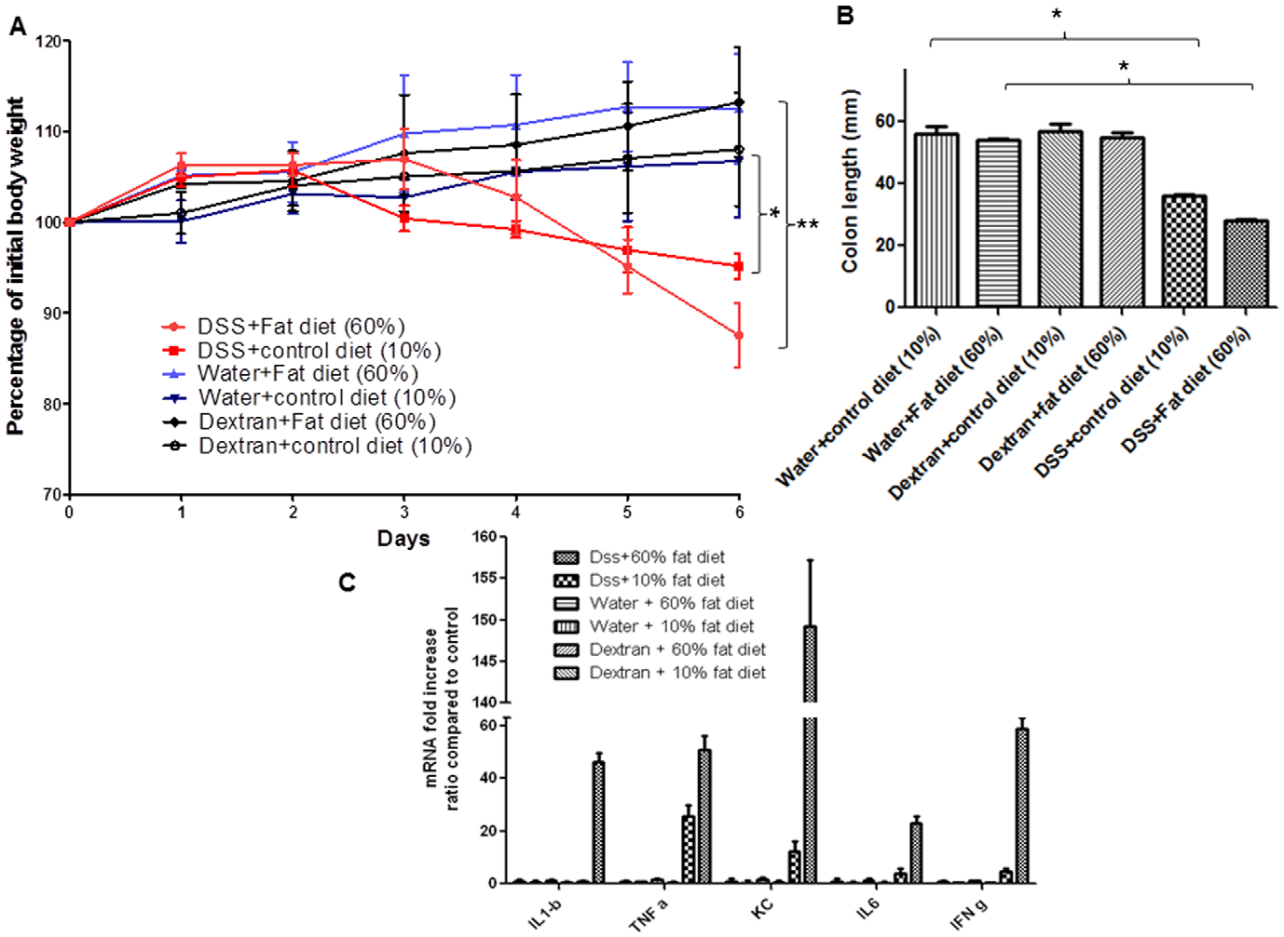
A high-fat diet increases the efficacy by which DSS induces colitis in mice. (A) Body weight changes in C57BL/6 mice on regular chow or a high-fat diet, drinking either a 3% (w/v) dextran or a 3% (w/v) DSS solution, for 6 days. Body weight changes are shown as means±SEMs. * P<0.05, ** P<0.01. (B) Determination of colon length (an index of colonic inflammation). Results are expressed in mm and represent means±SEMs of three independent determinations. * P<0.05. (C) Consumption of DSS and a high-fat diet significantly increases production of pro-inflammatory cytokines and chemokine mRNAs compared to what is seen in animals consuming DSS but (otherwise) regular chow. * P<0.05, ** P<0.01, *** P<0.001.

The above experiments showed that DSS did not interact directly with epithelial cells via a specific receptor, nor was inflammation induced by sub-products generated from DSS by bacterial degradation/modification. We next explored whether a high fat diet affected the efficacy of DSS in terms of inducing colitis in mice. As shown in Figure 6, ingestion of 3% (w/v) DSS and a high-fat diet (60% [w/w] fat) worsened colitis, as assessed by weight loss (Figure 6A), compared to use of DSS and the normal mouse chow (<10% fat). The observed effect was caused by the DSS/fatty diet combination because mice drinking regular drinking water and eating the high-fat diet showed no significant weight loss compared to mice with access to regular drinking water and regular chow after 6 days of treatment (Figure 6A). Also, ingestion of the high-fat diet increased the inflammation seen after DSS treatment compared to that noted when DSS was given together with regular diet. Thus, comparing the mice given a high-fat diet and DSS versus a regular diet coupled with DSS, the colon length was further reduced (28 mm versus 36 mm in Figure 6B) and pro-inflammatory cytokine expression levels were increased (46-fold increase in IL-1β mRNA level; 50-fold versus 25-fold in TNFα mRNA level; 23-fold versus 3.7-fold in IL6 mRNA level; 58.6-fold versus 4.7-fold in INFγ mRNA level; and 150-fold in KC mRNA level versus 10-fold in mice given regular chow) (Figure 6C). Interestingly, the high-fat diet acted in a manner similar to a cofactor, exacerbating the effect of DSS. These results were not observed when mice had access to regular water and a high-fat diet (Figure 6; A, B, and C). The results suggest that fatty acids specifically exacerbated DSS-induced colitis in mice. We propose that a potential electrostatic association between fatty acids and DSS, mediated via dicationic ions (Ca^++^, Mg^+^) or positively charged molecules, could result in formation of DSS-loaded vesicles which, via the hydrophobic domains, might fuse with the membranes of colonic cells and subsequently deliver DSS into such cells. Once in the cytosol, DSS would activate pro-inflammatory cascades. Several examples of inflammation caused by DSS have been reported. Interestingly, Miyazawa et al. [23] showed that DSS caused disruption of biological mechanisms (such as inhibitory effects on reverse transcriptase activities that affect major cellular functions), competing with poly(U) to this end [23]. Previously, it was shown that dextran sulfate inhibited ribonuclease action [24], [25]. Other natural and synthetic polyanionic polymers play important roles in establishing the association of mRNA with ribosomes and can disturb mRNA translation [23].

### DSS associated with fatty acids disrupts Caco2-BBE monolayer resistance

**Figure 7:**
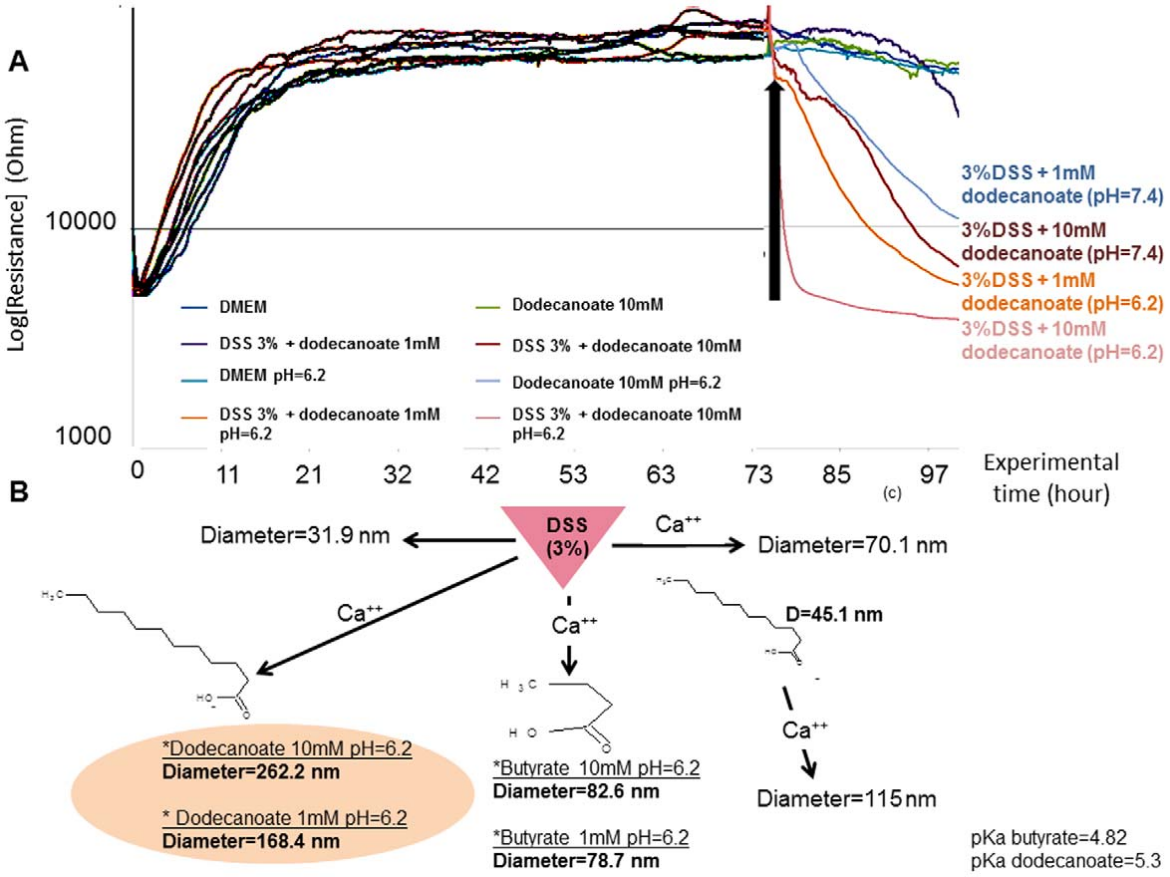
DSS and fatty acids form nano-lipocomplexes that disrupt Caco2-BBE monolayer resistance. (A) Confluent cultures were obtained after 48 h of growth and epithelial resistance was assessed by continuous resistance measurement at pH 7.4 (cellular pH) or pH 6.2 (colonic pH). The epithelial resistances of cultures treated with dodecanoate alone (10 mM) or with 3% (w/v) DSS (1 or 10 mM dodecanoate), at pH 6.2 or pH 7.4, were assessed by ECIS. (B) Light scattering measurement of the diameters (nm) of particles formed by DSS (3% w/v) alone; DSS (3% w/v) with Ca^++^ (1 mM); and DSS (3% w/v) with Ca^++^ (1 mM) and butyrate (1 or 10 mM) or dodecanoate (1 or 10 mM).

Using ECIS, we showed that 3% (w/v) DSS did not affect *in vitro* intestinal barrier function (Figure 4). We also checked that the supplementation of the medium with 5% fetal bovine serum (FBS) had no effect on the epithelial barrier disruption (data not shown). Next, we investigated the effects of fatty acids (dodecanoate and butyrate salts) on the ability of DSS to disrupt *in vitro* intestinal barrier function. Caco2-BBE monolayer resistance in DMEM at pH 7.4 (cellular pH) and pH 6.2 (colonic pH) was assessed by ECIS. Butyrate did not significantly affect resistance (data not shown). As shown in Figure 7A, dodecanoate alone (10 mM) had no effect on epithelial resistance. However, addition of 3% (w/v) DSS supplemented with dodecanoate (10 mM, pH 6.2) to Caco2-BBE monolayers caused a rapid decrease (89%) in monolayer resistance, from 47,790 Ω to 5,420 Ω after 3 h (Figure 7A). Interestingly, under the same conditions but at pH 7.4, a slower and smaller decrease in resistance was observed (a 77% decrease after 23 h). This may be explained by variation in the hydrophobicity of vesicles at pH 7.4 and pH 6.2. Vesicles at pH 6.2 carry fewer negative charges than at pH 7.4 and can therefore engage in a higher level of interaction with the plasma membrane, which is generally negatively charged.

Next, we explored whether a mixture of DSS and fatty acids could form vesicles. DSS mixed with dodecanoate but not butyrate, in the presence of calcium, formed nanometer-sized vesicles (Figure 7B) 262 nm and 168 nm in diameter, respectively, when dodecanoate was used at 10 mM and 1 mM. No other DSS/fatty acid combination tested formed vesicles. Smaller vesicles were formed when DSS was complexed with calcium only (diameter 70 nm) or butyrate only (115 nm). Our data suggest that DSS (likely via the sulfate groups) engages in electrostatic interactions with carboxylate groups of medium or long fatty acids, involving bridging by Ca^++^ ions. Once vesicles are formed, they fuse with cell membranes and deliver DSS into the cytoplasm.

### Dextran-loaded nanoparticles induce colitis

**Figure 8:**
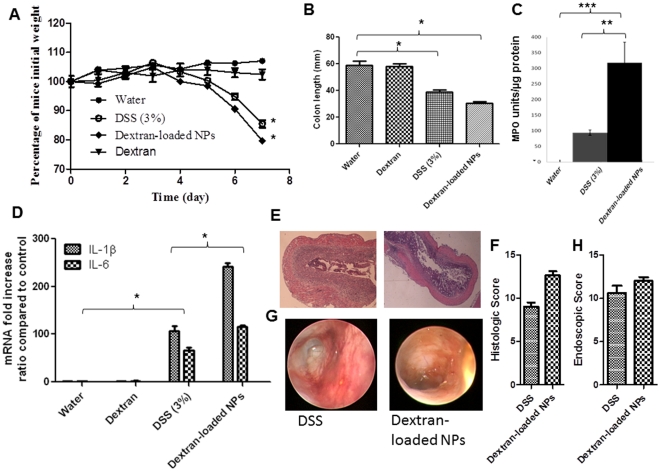
Dextran-loaded nanoparticles but not free dextran induce colitis. Mouse body weight changes during treatment with DSS supplemented with dextran-loaded NPs (“dextran-loaded NPs”) or empty NPs (“DSS 3%”) compared to those of control mice gavaged with empty NPs suspended in water. C57BL/6 mice drank a 3% (w/v) DSS solution, or water, for the indicated numbers of days. (A) Body weight changes are shown as means±SEMs. * P<0.05. (B) Colon length (mm) of DSS-treated mice given dextran-loaded or empty NPs, compared to that of control animals (drinking water only). * P<0.05. (C) Determination of MPO enzymatic activity as an index of neutrophil infiltration into injured tissue. Results are expressed as units of MPO per µg of protein, are compared to the values in control mice, and are means±SEMs of three independent determinations. ** P<0.01, *** P<0.001. (D) Dextran-loaded NPs can significantly increase production of IL-1β and IL-6, compared to use of empty NPs, in animals drinking DSS (3% w/v). mRNA expression was measured in test and control animals. * P<0.05. (E) Hematoxylin-stained colon sections of mice receiving daily gavage with dextran-loaded or empty NPs (“DSS”) in the interval during which the drinking water contained DSS (3% w/v); thus on day 7. (F) Histological scores. (G) Macroscopic inflammation was assessed using a mouse colonoscope. Photographs were obtained on the day of sacrifice and, (H), endoscopic scores were calculated.

We thus showed that DSS/dodecanoate formed nanometer-sized vesicles taken up by Caco2-BBE monolayers. We speculated that the limiting step in *in vivo* colitis development induced by DSS was formation of nanometric vesicles via association of DSS and fatty acids. As dextran-loaded lipid vesicles are difficult to deliver to the colon, we decided to investigate the effects of dextran-loaded nanoparticles (NPs) *in vivo*. In previous studies, we have shown that we can deliver such NPs into the colon [39], [40], [42], [46]. We chose to deliver dextran to the colon because dextran does not form vesicles in the presence of dodecanoate and does not induce colitis *in vivo*. We found that dextran-loaded NPs, but not free dextran, given by gavage over 8 days, increased measures of inflammatory parameters, such as weight loss (at day 7, 23% for mice given dextran-loaded NPs versus 17% for those given 3% [w/v] DSS) (Figure 8A), decreased colon length (Figure 8B) (to 28 mm versus 38 mm for DSS-treated mice), increased MPO activity (Figure 8C) (312 MPO units/µg protein for dextran-loaded nanoparticle-treated mice versus 96 MPO units/µg protein for DSS-treated mice), and increased the levels of pro-inflammatory cytokines (Figure 8D) (2.6-fold increase in IL-1β level and a 1.5-fold increase in IL-6 level for mice treated with NPs versus DSS alone). Again, all of these observations were confirmed by histology and endoscopy (Figures 8E and 8F). Finally, induction of colitis was more severe using dextran-loaded NPs compared to DSS alone (Figure 8A to 8H). The results suggest that dextran may be the active motif of the DSS molecule in terms of induction of colitis.

## Discussion

During DSS treatment, inflammation is enhanced, as shown by rises in the extent of diarrhea and rectal bleeding. Colonic inflammation is also characterized by severe lesions throughout the mucosa, alteration of epithelial structure, high-level neutrophil and lymphocyte infiltration into the mucosal and submucosal areas, and loss of crypts. We explored how DSS, a negatively charged polymer of glucose with engrafted sulfate groups, induces colitis in mice. We tested different components of the molecule of DSS such as glucose, sulfate sodium, dextran and association of each. DSS-associated molecules had no proinflammatory effects. We did not observe any signs of inflammation (loss of barrier functions, apoptosis, etc.) *in vitro* in Caco2-BBE monolayer cells nor during *in vivo* study. Conversely, we showed that a high-fat diet increases the efficacy by which DSS induces colitis in mice. From those observations, we tested the effect of DSS on Caco2-BBE monolayer cells but with cell medium containing MCFAs (C12). We showed the ability of DSS associated with MCFAs to disrupt *in vitro* intestinal barrier function whereas butyrate (small chain fatty acid) did not significantly affect resistance. Next, we showed that DSS mixed with dodecanoate but not butyrate, in the presence of calcium, formed nanometer-sized vesicles.

It has been shown in the literature that claudins have significant interaction with MCFAs, and so could be involved in the “transport” of DSS through the epithelium, explaining the loss of membrane integrity shown in our article. *In vivo*, several MCFAs, particularly capric (C10) and lauric (C12) acids, have been shown to increase rectal drug absorption, presumably by causing alterations in intestinal TJ barrier function. C10 has also been shown to lead to profound alterations in the barrier function of the TJ and has been investigated as an agent to enhance viral-mediated gene transfer [27].

### Conclusion

In the present study we have made progress toward elucidating the mechanism of action of DSS in terms of induction of colitis. Our findings suggest that DSS associates with medium-chain-length fatty acids (MCFAs) such as dodecanoate in the colonic lumen prior to induction of colitis. Interestingly, it is known that MCFAs are present at high concentrations in the colonic lumen and that the colonic epithelium absorbs and partially metabolizes MCFAs [47]. DSS complexed to MCFAs form nanometer-sized vesicles ∼200 nm in diameter that fuse with colonocyte membranes. The arrival of such vesicles in the cytoplasm affects major epithelial cell pathways and consequently reduces intestinal barrier functions that initiate intestinal inflammatory signaling cascades. We also suggest that the inflammatory activity of DSS is afforded by the dextran moiety. This idea is supported by the fact that, when dextran is delivered to the cytoplasm, intestinal inflammatory signaling pathways are activated. The deleterious activity of dextran may be explained by the molecule having an inhibitory effect on reverse transcriptase activities that affect major cellular functions. The deleterious effects of DSS are targeted principally to the distal colon. It will be useful to chemically modify DSS molecules, rendering the molecule beneficial to the colon, without affecting targeting specificity.

## References

[1] Orholm M, Munkholm P, Langholz E, Nielsen OH, Sorensen TI (1991). Familial occurrence of inflammatory bowel disease.. N Engl J Med.

[2] Lowe AM, Roy PO, M BP, Michel P, Bitton A (2009). Epidemiology of Crohn's disease in Quebec, Canada.. Inflamm Bowel Dis.

[3] Brant SR (2011). Update on the heritability of inflammatory bowel disease: the importance of twin studies.. Inflamm Bowel Dis.

[4] Skewis LR, Reinhard BM (2010). Control of colloid surface chemistry through matrix confinement: facile preparation of stable antibody functionalized silver nanoparticles.. ACS applied materials & interfaces.

[5] Riva G, Wiederhold BK, Mantovani F, Gaggioli A (2011). Interreality: the experiential use of technology in the treatment of obesity.. Clinical practice and epidemiology in mental health : CP & EMH.

[6] Schlitt HJ, Mornex F, Shaked A, Trotter JF (2011). Immunosuppression and hepatocellular carcinoma..

[7] Magitta NF, Pura M, Wolff ASB, Vanuga P, Meager A (2008). Autoimmune polyendocrine syndrome type I in Slovakia: relevance of screening patients with autoimmune Addison's disease.. European journal of endocrinology/European Federation of Endocrine Societies.

[8] Hu Y, Mak JF, Lu WW, Cheung KM, Luk KD (2004). Visualization of lumbar muscle contraction synergy using surface electromyography (sEMG) streaming topography.. Conference proceedings : Annual International Conference of the IEEE Engineering in Medicine and Biology Society IEEE Engineering in Medicine and Biology Society Conference.

[9] Walsh DA, McWilliams DF (2006). Tachykinins and the cardiovascular system.. Current drug targets.

[10] Pinho RA, Silveira PC, Silva LA, Luiz Streck E, Dal-Pizzol F (2005). N-acetylcysteine and deferoxamine reduce pulmonary oxidative stress and inflammation in rats after coal dust exposure.. Environmental research.

[11] Toth G, R FM, Lovas S (2001). Stabilization of local structures by pi-CH and aromatic-backbone amide interactions involving prolyl and aromatic residues.. Protein engineering.

[12] Miyazawa F, Olijnyk OR, Tilley CJ, Tamaoki T (1967). Interactions between dextran sulfate and Escherichia coli ribosomes.. Biochimica et biophysica acta.

[13] Nakamura T, Katori R, Watanabe T, Miyazawa K, Murai M (1967). Quantitation of left-to-right shunt from a single earpiece dye-dilution curve.. Journal of applied physiology.

[14] Nakamura T, Katori R, Miyazawa K, Oda J, Ishikawa K (1967). Measurement of bronchial blood flow in tetralogy of Fallot.. Circulation.

[15] Yan Y, Kolachala V, Dalmasso G, Nguyen H, Laroui H (2009). Temporal and spatial analysis of clinical and molecular parameters in dextran sodium sulfate induced colitis.. PLoS One.

[16] Rogler G, Andus T (1998). Cytokines in inflammatory bowel disease.. World J Surg.

[17] Kennedy RJ, Hoper M, Deodhar K, Erwin PJ, Kirk SJ (2000). Interleukin 10-deficient colitis: new similarities to human inflammatory bowel disease.. Br J Surg.

[18] Steidler L, Hans W, Schotte L, Neirynck S, Obermeier F (2000). Treatment of murine colitis by Lactococcus lactis secreting interleukin-10.. Science.

[19] Hofstetter C, Kleen M, Habler O, Allmeling AM, Krombach F (1998). Recombinant human interleukin-10 attenuates TNFalpha production by porcine monocytes.. Eur J Med Res.

[20] Cooper HS, Murthy SN, Shah RS, Sedergran DJ (1993). Clinicopathologic study of dextran sulfate sodium experimental murine colitis.. Lab Invest.

[21] Cooper HS, Murthy SN, Shah RS, Sedergran DJ (1993). Clinicopathologic study of dextran sulfate sodium experimental murine colitis.. Laboratory investigation; a journal of technical methods and pathology.

[22] Dignass A, Preiss JC, Aust DE, Autschbach F, Ballauff A (2011). [Updated German Guideline on Diagnosis and Treatment of Ulcerative Colitis, 2011.].. Zeitschrift fur Gastroenterologie.

[23] Miyazawa F, Olijnyk OR, Tilley CJ, Tamaoki T (1967). Interactions between dextran sulfate and Escherichia coli ribosomes.. Biochim Biophys Acta.

[24] Fellig J, Wiley CE (1959). The inhibition of pancreatic ribonuclease by anionic polymers.. Arch Biochem Biophys.

[25] Philipson L, Zetterqvist O (1964). The Presence of DNA in Human Erythrocyte Membranes.. Biochim Biophys Acta.

[26] Kim IW, Myung SJ, Do MY, Ryu YM, Kim MJ (2010). Western-style diets induce macrophage infiltration and contribute to colitis-associated carcinogenesis.. Journal of gastroenterology and hepatology.

[27] Coyne CB, Kelly MM, Boucher RC, Johnson LG (2000). Enhanced epithelial gene transfer by modulation of tight junctions with sodium caprate.. American journal of respiratory cell and molecular biology.

[28] Lindmark T, Nikkila T, Artursson P (1995). Mechanisms of absorption enhancement by medium chain fatty acids in intestinal epithelial Caco-2 cell monolayers.. The Journal of pharmacology and experimental therapeutics.

[29] Lindmark T, Soderholm JD, Olaison G, Alvan G, Ocklind G (1997). Mechanism of absorption enhancement in humans after rectal administration of ampicillin in suppositories containing sodium caprate.. Pharmaceutical research.

[30] Tomita M, Hayashi M, Awazu S (1995). Absorption-enhancing mechanism of sodium caprate and decanoylcarnitine in Caco-2 cells.. The Journal of pharmacology and experimental therapeutics.

[31] Tomita M, Hayashi M, Awazu S (1996). Absorption-enhancing mechanism of EDTA, caprate, and decanoylcarnitine in Caco-2 cells.. Journal of pharmaceutical sciences.

[32] Andoh A, Bamba T, Sasaki M (1999). Physiological and anti-inflammatory roles of dietary fiber and butyrate in intestinal functions.. JPEN Journal of parenteral and enteral nutrition.

[33] Venkatraman A, Ramakrishna BS, Pulimood AB, Patra S, Murthy S (2000). Increased permeability in dextran sulphate colitis in rats: time course of development and effect of butyrate.. Scandinavian journal of gastroenterology.

[34] Venkatraman A, Ramakrishna BS, Shaji RV, Kumar NS, Pulimood A (2003). Amelioration of dextran sulfate colitis by butyrate: role of heat shock protein 70 and NF-kappaB.. American journal of physiology Gastrointestinal and liver physiology.

[35] Vieira EL, Leonel AJ, Sad AP, Beltrao NR, Costa TF (2011). Oral administration of sodium butyrate attenuates inflammation and mucosal lesion in experimental acute ulcerative colitis.. The Journal of nutritional biochemistry.

[36] Charrier L, Yan Y, Driss A, Laboisse CL, Sitaraman SV (2005). ADAM-15 inhibits wound healing in human intestinal epithelial cell monolayers.. Am J Physiol Gastrointest Liver Physiol.

[37] Laroui H, Grossin L, Leonard M, Stoltz JF, Gillet P (2007). Hyaluronate-covered nanoparticles for the therapeutic targeting of cartilage.. Biomacromolecules.

[38] Lamprecht A, Yamamoto H, Takeuchi H, Kawashima Y (2005). A pH-sensitive microsphere system for the colon delivery of tacrolimus containing nanoparticles.. J Control Release.

[39] Laroui H, Dalmasso G, Nguyen HT, Yan Y, Sitaraman SV (2010). Drug-loaded nanoparticles targeted to the colon with polysaccharide hydrogel reduce colitis in a mouse model.. Gastroenterology.

[40] Laroui H, Theiss AL, Yan Y, Dalmasso G, Nguyen HT (2011). Functional TNFalpha gene silencing mediated by polyethyleneimine/TNFalpha siRNA nanocomplexes in inflamed colon.. Biomaterials.

[41] Vowinkel T, Kalogeris TJ, Mori M, Krieglstein CF, Granger DN (2004). Impact of dextran sulfate sodium load on the severity of inflammation in experimental colitis.. Dig Dis Sci.

[42] Theiss AL, Laroui H, Obertone TS, Chowdhury I, Thompson WE (2011). Nanoparticle-based therapeutic delivery of prohibitin to the colonic epithelial cells ameliorates acute murine colitis.. Inflamm Bowel Dis.

[43] Shea-Donohue T, Thomas K, Cody MJ, Aiping Z, Detolla LJ (2008). Mice deficient in the CXCR2 ligand, CXCL1 (KC/GRO-alpha), exhibit increased susceptibility to dextran sodium sulfate (DSS)-induced colitis.. Innate Immun.

[44] te Velde AA, de Kort F, Sterrenburg E, Pronk I, ten Kate FJ (2007). Comparative analysis of colonic gene expression of three experimental colitis models mimicking inflammatory bowel disease.. Inflamm Bowel Dis.

[45] Mitsuyama K, Tsuruta O, Tomiyasu N, Takaki K, Suzuki A (2006). Increased circulating concentrations of growth-related oncogene (GRO)-alpha in patients with inflammatory bowel disease.. Dig Dis Sci.

[46] Laroui H, Wilson DS, Dalmasso G, Salaita K, Murthy N (2011). Nanomedicine in GI.. Am J Physiol Gastrointest Liver Physiol.

[47] Schmidt C, Dignass A, Hartmann F, Huppe D, Kruis W (2011). [IBD Ahead 2010 - Answering Important Questions in Crohn's Disease Treatment].. Zeitschrift fur Gastroenterologie.

